# Lowering of Proton and Deuteron Mean Kinetic Energy in the LiTFSI Water-in-Salt Electrolyte System

**DOI:** 10.1021/acs.jpclett.5c03900

**Published:** 2026-02-03

**Authors:** Mi Zhang, Andrew G. Seel, Patrick L. Cullen

**Affiliations:** † School of Engineering and Materials Science, 4617 Queen Mary University of London, London E1 4NS, U.K.; ‡ ISIS Spallation Neutron and Muon Source, 97008 Rutherford Appleton Laboratory, Didcot OX11 0QX, U.K.; ¶ Department of Physics, Royal Holloway University of London, Egham TW20 0EX, U.K.

## Abstract

Water-in-salt electrolytes have emerged as promising materials for energy storage devices, significantly extending the electrochemical stability window of water through confinement within a salt matrix. While the structure and distribution of water molecules in these systems is becoming increasingly better characterized, the molecular nature and energetics of water present a greater challenge. Measurement of the quantum kinetic energy of light atoms is a sensitive probe of their environment, reflecting the potential experienced by the atoms and inclusive of their zero-point energy. It is found that the mean kinetic energy of the proton and deuteron in the archetypal water-in-salt electrolyte system, LiTFSI-H_2_O, decreases as a function of salt concentration. This indicates that while the disruption of the hydrogen bond network of water is known to lead to an increasing OH stretching frequency, other components to the quantum kinetic energy must decrease to result in the overall lower measured average.

## Graphical abstract

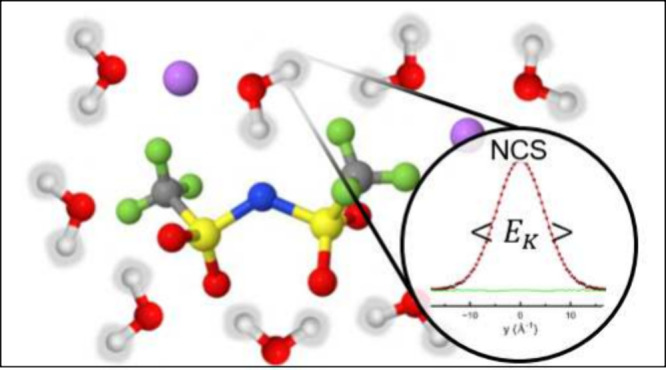

Water-in-salt electroyte (WiSE) systems are a family of highly concentrated aqueous solutions of various s-block or combinations of s- and d-block metals, currently of great interest due to their proposed applications in battery and energy technologies.^1–4^ Water as an electrolyte possesses a number of proposed technological benefits, such as recyclability and green-chemical properties, abundance, and low cost, but is limited by having a narrow electrochemical stability window. This is circumvented in WiSE systems due to the ability to reach concentrations up to 21 M having the effect of raising the breakdown voltage of water, thereby bringing aqueous solutions into the realm of lithium ion electrolytes.

Water is, of course, also an extremely important and fundamental compound across the physical and biological sciences. The nature of the ambient liquid and other condensed phases of water has been and continues to be the focus of much experimental and theoretical study, in particular regarding the extent and effect of hydrogen bonding or its absence on the chemical state of water within different confining media. A powerful probe of the state of the proton in water is neutron Compton scattering (NCS),^5,6^ also referred to in the literature as deep inelastic neutron scattering (DINS). This technique enables a direct experimental determination of the mean proton kinetic energy, $\left\langle E_{K}\right\rangle$ , including zero-point energy, rather than, for example, examining transitions between various vibrational states as performed by inelastic neutron scattering, infrared absorption, or Raman scattering techniques. As such, a number of NCS studies have been performed on liquid water,^7–11^ supercooled and supercritical water,^12–14^ and water in various confining media such as xerogels,^15^ silica,^16,17^ membrane materials,^18^ and under the application of pressure.^19,20^

In this study, we report the results of NCS measurement on an archetypal WiSE system, LiTFSI-H_2_O, across a wide concentration range, with the aim of determining how the dissolution of water within the liquid salt network affects the mean proton kinetic energy. Dried LiTFSI salts were handled under an inert atmosphere before being dissolved in stoichiometric amounts of either H_2_O or D_2_O. Concentrations are given in terms of salt molality in H_2_O. Neutron Compton scattering measurements were performed using the VESUVIO spectrometer at the ISIS Spallation Neutron and Muon Source.^21^ Proteated samples were measured for a period of approximately 24 hr (equivalent to over 3000 μAh) and deuterated samples were measured for 36 hr (>4500 μAh). Samples were measured at 300 K in flat plate geometry with sample thicknesses varied from 0.5 mm to 2 mm depending on concentration and scattering power in order to minimize the effects of multiple scattering. Data were reduced and corrected for instrumental background, multiple scattering, and final-state effects using the standard reduction routines of VESUVIO.^22^ Within the impulse approximation the scattering response function, *S*(**Q**, ω), can be recast in terms of single atom scattering using the scaling variable *y* = $\frac{M}{\hbar Q}\left(\omega -\frac{\hbar Q^{2}}{2M}\right)$ :^5,6^

1 JM(y,Q̂)=QMS(Q,ω)=∫n(p)δ(y−Q̂·p)dp

where Q̂ is the unit scattering vector and *n*(**p**) is the momentum distribution for the scattering atom of mass *M*. Due to the isotropic nature of the liquid WiSE samples, *J*_*M*_(*y*) has been modeled as taking an isotropic Gaussian form for all masses:

2 $$J_{M}\left(y\right)=\frac{1}{\sqrt{2\pi \sigma_{M}^{2}}}\exp\left(\frac{-y^{2}}{2\sigma_{M}^{2}}\right)$$

The standard deviation of *J*_*M*_(*y*) relates to the mean kinetic energy of the scattering atom, $\langle E_{K}\rangle =3\hbar \sigma_{M}^{2}/2M$ . The inclusion of anharmonic terms to extend *J*_*M*_(*y*) for the proton or deuteron were not found to improve fits to the experimental data significantly and were deemed unnecessary considering the distribution of chemical environments of the water hydrogen in these multicomponent WiSE systems. NCS spectra were collected for LiTFSI WiSE solutions for both proteated and deuterated water analogues, henceforth termed H-WiSE and D-WiSE. Representative spectra for samples of 1 M concentration are shown in Figure 1. It can be readily seen that the spectral weight of the proton or deuteron are distinct from that of other masses in the system (each of the water O and the atoms in LiTFSI), with time-of-flight (ToF) values depending both on the mass of the recoiling atom and the detector position as shown in the left-hand panels. The proton intensity is far greater than that of deuterium due to the larger neutron scattering cross-section of the former (σ_*H*_ = 82.02b, σ_*D*_ = 7.64b). As discussed above and detailed elsewhere, the coupling of wavevector- and energy-transfer in the impulse regime of NCS allows for every unique ToF spectra to be collapsed onto the same, mass-specific momentum space, so-called *y*_*M*_-scaling.^5,6^ The proton and deutron momentum profiles, *J*(*y*), are shown in the right-hand panels.

**Figure 1:**
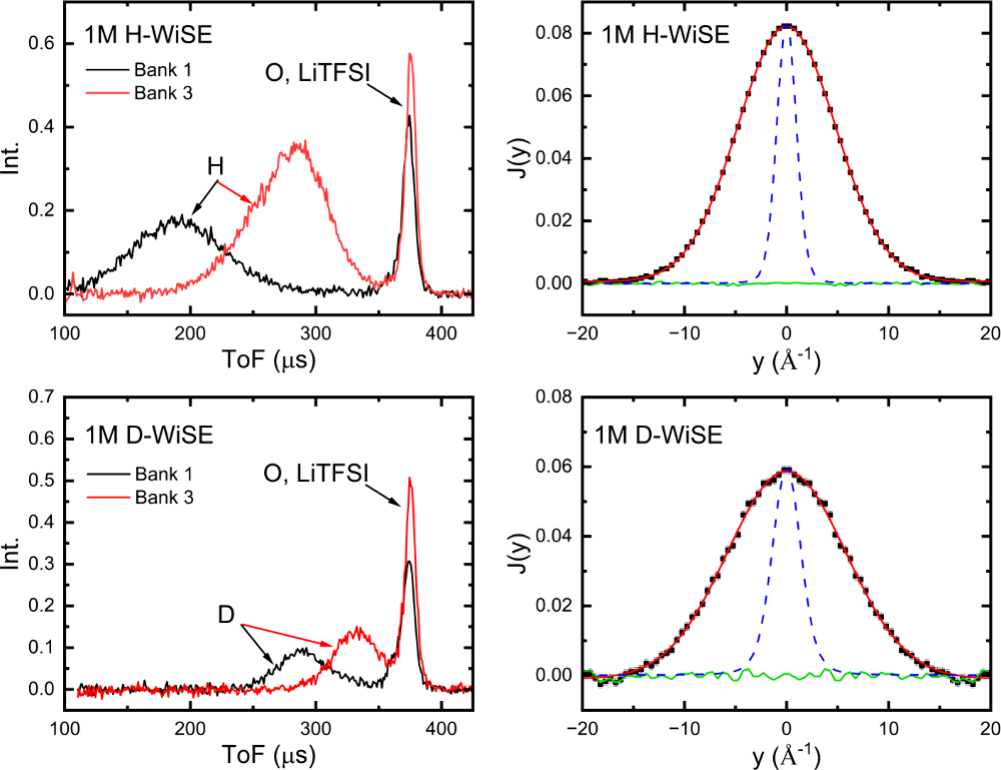
Representative NCS data for 1 M LiTFSI WiSE. *Left panels*: ToF spectra for two detector banks with nominal angles of 65° (bank 1) and 45° (bank 3). *Right panels*: Data (black), fitted (red), and residual (green) Compton profiles for the proton/deuteron for all detectors transformed to *J*(*y*). The blue dashed line is the instrumental resolution in *y*-space for each mass.

The measured values σ_*M*_ for both H-WiSE and D-WiSE samples are presented in Table 1, and corresponding $\left\langle E_{K}\right\rangle$ values are shown in Figure 2 across a wide concentration range. The ratio of σ_*M*_ values is within an error of $\sqrt[{4}]{2}$ expected within a harmonic system, although this is overshadowed by the relatively large error for the deuterated samples due to the scattering power and kinematics of deuterium measurements. This ratio should not be taken as particularly significant and serves simply as an initial check on the data validity. Moving on to the evaluated $\left\langle E_{K}\right\rangle$ values and first examining the proteated system, we see that $\left\langle E_{K}\right\rangle$ decreases with concentration but is constant above 10 m within error. There is an overall drop of approximately 10% in the value of $\left\langle E_{K}\right\rangle$ . The deuterated samples also demonstrate an overall decrease in $\left\langle E_{K}\right\rangle$ and earlier plateauing within the aforementioned higher error.

**Figure 2:**
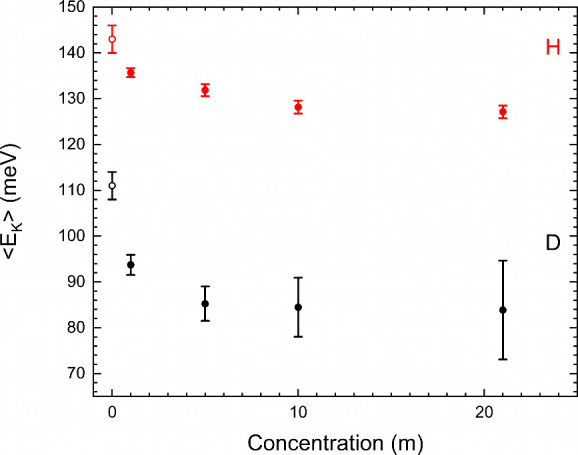
Mean kinetic energy values for the proton (red, top) and deuteron (black, bottom) for LiTFSI WiSE samples at different concentrations. Filled circles are from the current study; open circles are taken from previous studies.^10,11^

**Table 1:** Measured Profile Widths, *σ*_*M*_, for the Proton and Deuteron in LiTFSI WiSE Solutions at Various Concentrations

Conc. (M)	σ_*H*_ (Å^–1^)	σ_*D*_ (Å^–1^)	σ_*D*_/σ_*H*_
1	4.669 ± 0.017	5.487 ± 0.065	1.175 ± 0.082
5	4.602 ± 0.023	5.232 ± 0.116	1.137 ± 0.139
10	4.537 ± 0.025	5.209 ± 0.199	1.148 ± 0.224
21	4.519 ± 0.024	5.190 ± 0.334	1.148 ± 0.358

The lowering of $\left\langle E_{K}\right\rangle$ for WiSEs is interesting, as it is significantly more pronounced that found previously for NaCl solutions or for multisalt solutions at ambient pressures.^20^ In these cases the lowering of $\left\langle E_{K}\right\rangle$ for the proton was found to be of a few rather than several  meV, and AIMD/PIMD simulations for dilute salt solutions have also suggested a decreasing $\left\langle E_{K}\right\rangle$ for water coordinated around both cation (Na^+^) and anion (Cl^–^, ${{\mathrm{HPO}}_{4}}^{2-}$ ).^23^ The effects of the larger anion solvation were more significant than those of the monatomic ions, which is an important consideration for WiSE systems. Certainly the lowering of $\left\langle E_{K}\right\rangle$ with concentration in this study indicates a stabilization of the average proton ground state of the water molecules in WiSEs, although these measurements cannot directly probe whether this contributes to the exceptional electrochemical stability widow in these systems. The underlying origin of the decrease in $\left\langle E_{K}\right\rangle$ has been attributed to competing effects of vibrational and rotational/translational degrees of freedom. To clarify further, moving from bulk water to the case of an individual water molecule (as in the gas-phase), the vibrational kinetic energy increases whereas the rotational and translational components both decrease, despite the absolute value of the kinetic energy of water being dominated by zero-point motion and raised far above the classical equipartition value. A similar effect has been predicted for the introduction of salts in solution although experimental evidence for this is still limited.^20,23^ An interesting result from these simulations is that, although cations are predicted to increase the vibrational kinetic energy for water molecules in the primary solvation sphere, something well documented albeit indirectly by an increase in OH stretching frequencies, the case of anions depends on their size and coordination ability to the water. In both cases, there is still a compensation between vibrational and translational/rotational degrees of freedom: as one component increases/decreases, the others decrease/increase. This effect carries into the secondary solvation to decrease the overall $\left\langle E_{K}\right\rangle$ .

We can consider whether our WiSE data agrees with the behavior predicted above, and first note that the extremely high concentrations available for WiSE systems should accentuate trends compared to dilute solutions. We do indeed find an overall larger drop in $\left\langle E_{K}\right\rangle$ for both H-WiSE and D-WiSE systems and can interpret this as likely originating from the drastic changes in microstructure as concentration increases. While the existence and general concentration dependence of water domains in WiSEs have been extensively studied by small-angle X-ray and neutron studies,^24^ recent wide-angle neutron scattering studies have demonstrated that chain-like microdomains of fewer than 10 water molecules dominate the LiTFSI WiSE system at these concentrations.^25^ The dimensions of these domains were found to be constant above 10 m, which is in accordance with the apparent plateauing of $\left\langle E_{K}\right\rangle$ above this value.

Within this model of a microdomained water network, we can further pinpoint the origin of the lowered kintic energy by considering what is known about the vibrational state of water in WiSE systems. It is now well-documented that the OH stretching frequecy in WiSE solutions increases as a function of concentration, again with an apparent plateuing above 10 M.^4,26^ This apparent stiffening of the OH/OD bond as the hydrogen-bonding network of water is disrupted in WiSEs would act to increase $\left\langle E_{K}\right\rangle$ , were it the only change in water dynamics. As discussed above, however, the decreased hydrogen-bonding network must soften the perpendicular vibrational dynamics in order to lower the overall vibrational kinetic energy. This region of the vibrational spectra would coincide with various excitations of the TFSI^–^ anions, but our results suggest further inelastic neutron measurements around the water libration region may be fruitful. A softening of the perpendicular vibrational dynamics accompanying a decrease in translational and/or rotational kinetic energies explains the overall lowering of $\left\langle E_{K}\right\rangle$ as found in this study.

## Supplementary Material



## Notes

Transparent Peer Review report available (PDF)
