# Supplementary material for: Lowering of Proton and Deuteron Mean Kinetic Energy in the LiTFSI Water-in-Salt Electrolyte System
Source: J Phys Chem Lett. 2026 Feb 3;17(7):1978–81. doi: 10.1021/acs.jpclett.5c03900 (PMC12927015; doi:10.1021/acs.jpclett.5c03900)
Supplement: Supplementary file 1 [file jz5c03900_si_001.pdf]

jz-2025-03900a.R1

Name: Peer Review Information for "Lowering of Proton and Deuteron Mean Kinetic Energy in the LiTFSI Water-in-salt Electrolyte System"

First Round of Reviewer Comments

Reviewer: 1

Comments to the Author

Water-in-salt electrolytes (WiSE) are of significant interest due to their importance for both fundamental understanding of liquids and solutions, and their potential practical applications. It can offer new insights into liquid nanostructure, liquid dynamics, and thermodynamics, which all contributes to our ability to design functional electrolyte solutions. As authors have pointed out, the bulk nanostructure of LiTFSI WiSE is now relatively well understood due to extensive studies over the past decade. However, molecular dynamics and mass transport in such concentrated electrolyte remains elusive due to its complexity. This study uses deep inelastic neutron scattering to provide unique insights regarding the mean kinetic energy of hydrogen at different LiTFSI concentrations. This information is extremely difficult to access with other experimental techniques. I believe it is suitable to be published on JPCL after the authors have considered the following:

1. It is not clear in the current main text how the mean kinetic energy of hydrogen atoms is related to any structural or dynamic properties of WiSE. It is likely to be related to mass transport and the surprisingly large electrochemical stability window of WiSE, but the present discussion is too brief.

2. The difference in mean kinetic energy between proton and deuteron are consistently larger in electrolytes than in pure water at all salt concentrations. Can this provide any information regarding the water H-bond environment in WiSE vs bulk water? Especially given the presence of small chain-like microdomains according to diffraction studies.

3. The decrease in mean kinetic energy with salt concentration is attributed to the competing effects of vibrational and rotationa/translational modes. Can this be disentangled by other experiments or studies?

4. Author suggested that the mean kinetic energy plateaued above 10 M which is consistent with a number of other studies, however, from Figure 2, it seems to have plateaued above 5 M (within error).

5. For such high salt concentration, molality (m) is typically used to specify the solution concentration whereas the present study is based on molarity (M). Is this a typo or is the solution prepared based on moles of LiTFSI per litre of water? Please correct/clarify in the main text.

6. Page 2, line 7, missing unit? "Samples were measured at 300..."

Author's Response to Peer Review Comments:

Dr. Andrew Seel

ISIS Spallation Neutron and Muon Source

Rutherford Appleton Laboratory

OX11 0QX

13/01/26

Editorial Office

The Journal of Physical Chemistry Letters

Dear Editor,

On behalf of all authors, I would like to thank our reviewers for such a rapid response and for recommending our manuscript "*Lowering of proton and deuteron mean kinetic energy in the LiTFSI Water-in-salt electrolyte system*" for publication in the Journal of Physical Chemistry Letters. Please find attached alterations in response to the reviewers comments:

Reviewer: Page 2 line 7, missing unit

The absent unit of temperature has been added.

Reviewer: For such high concentration, molality (m) is typically used … please correct/clarify in the main text

Clarification has now been added on page 2 prior to discussion of the NCS technique: Dried LiTFSI salts were handled under an inert atmosphere before being dissolved in stoichiometric amounts of either H_2O or D_2O. Concentrations are given in terms of salt molality in \ch{H2O}.

Reviewer: Author suggested that mean kinetic energy plateaued above 10 m … however from Figure 2 is seems to have plateaued above 5 m

Additional clarification for the apparent earlier plateauing for the deuterated samples has been added to the main text: The deuterated samples also demonstrate an overall decrease in $\langle E_{!} \rangle$ and earlier plateauing within the aforementioned higher error.

Reviewer: The decrease in mean kinetic energy with salt contribution is attributed to the competing effects of vibrational and rotational/translational modes. Can this be disentangled by other experiments or studies?

Whilst this is perhaps speculative as it does not directly relate to the results reported in this study, we have provided additional discussion without our concluding remarks to this effect: As is discussed above, however, the decreased hydrogen-bonding network must soften the perpendicular vibrational dynamics in order to lower the overall vibrational kinetic energy. This region of the vibrational spectra would coincide with various excitations of the $TFSI^-$ anions, but our results suggest further inelastic neutron measurements around the water libration region may be fruitful. A softening of the perpendicular vibrational dynamics accompanying a decrease in translational and/or rotational kinetic energies explains the overall lowering of $\langle E_{!} \rangle$ as found in this study.

Reviewer: The difference in mean kinetic energy between proton and deuteron are consistently larger in electrolytes than in pure water at all salt concentrations. Can this provide any information regarding the water H-bond environment in WiSE vs bulk water? It is not clear in the current text how the kinetic energy of hydrogen atoms is related to structural or dynamical
